# Supplementary material for: Oxidation induced strain and defects in magnetite crystals
Source: Nat Commun. 2019 Feb 11;10:703. doi: 10.1038/s41467-019-08470-0 (PMC6370877; doi:10.1038/s41467-019-08470-0)
Supplement: Supplementary file 1 — Supplementary Information [file 41467_2019_8470_MOESM1_ESM.pdf]

Oxidation induced strain and defects in magnetite crystals

Yuan et al.

Supplementary Information

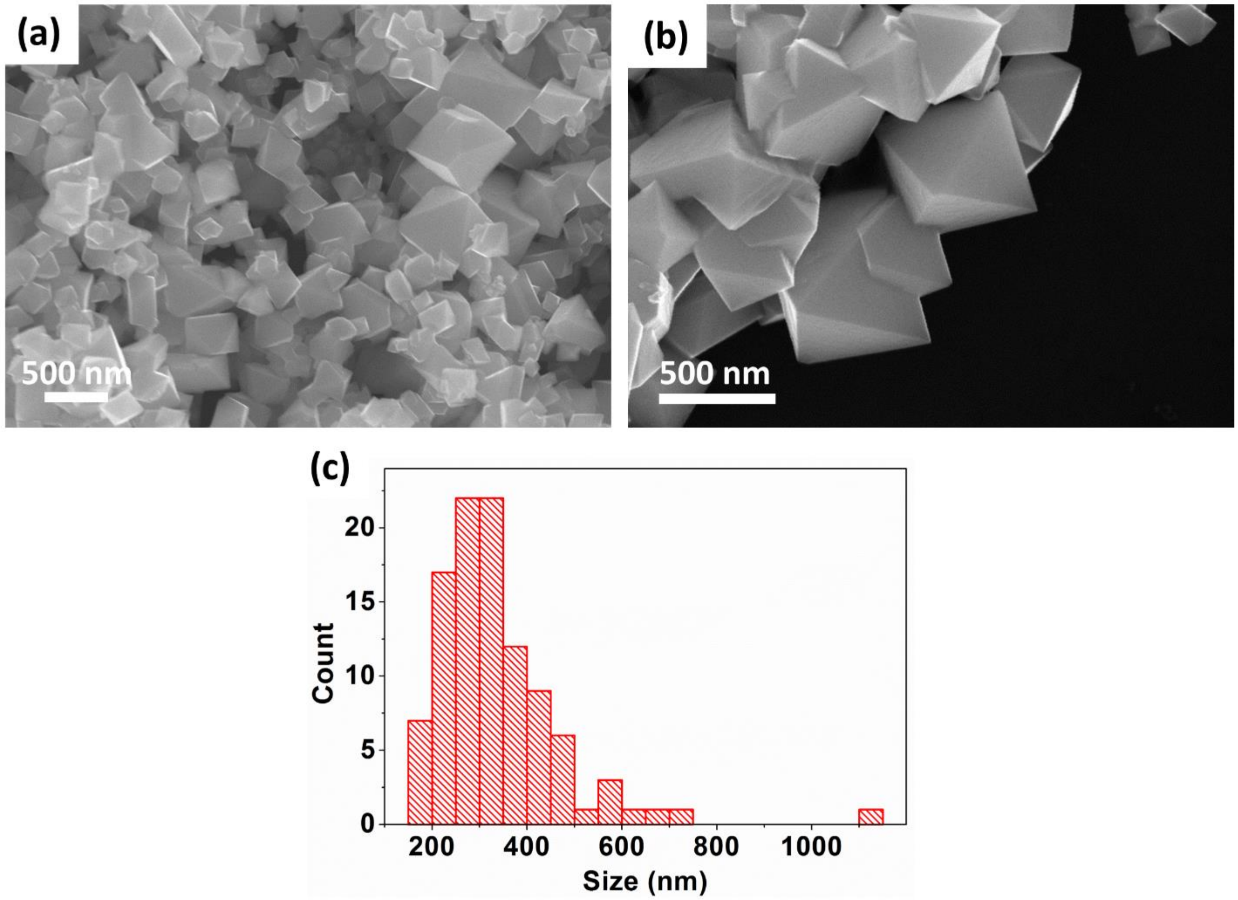

Supplementary Figure 1. **Morphology and size distribution of magnetite crystals.** (a, b) Scanning electron microscopy images of pristine magnetite crystals used for BCDI measurements. (c) Particle size distribution obtained from (a).

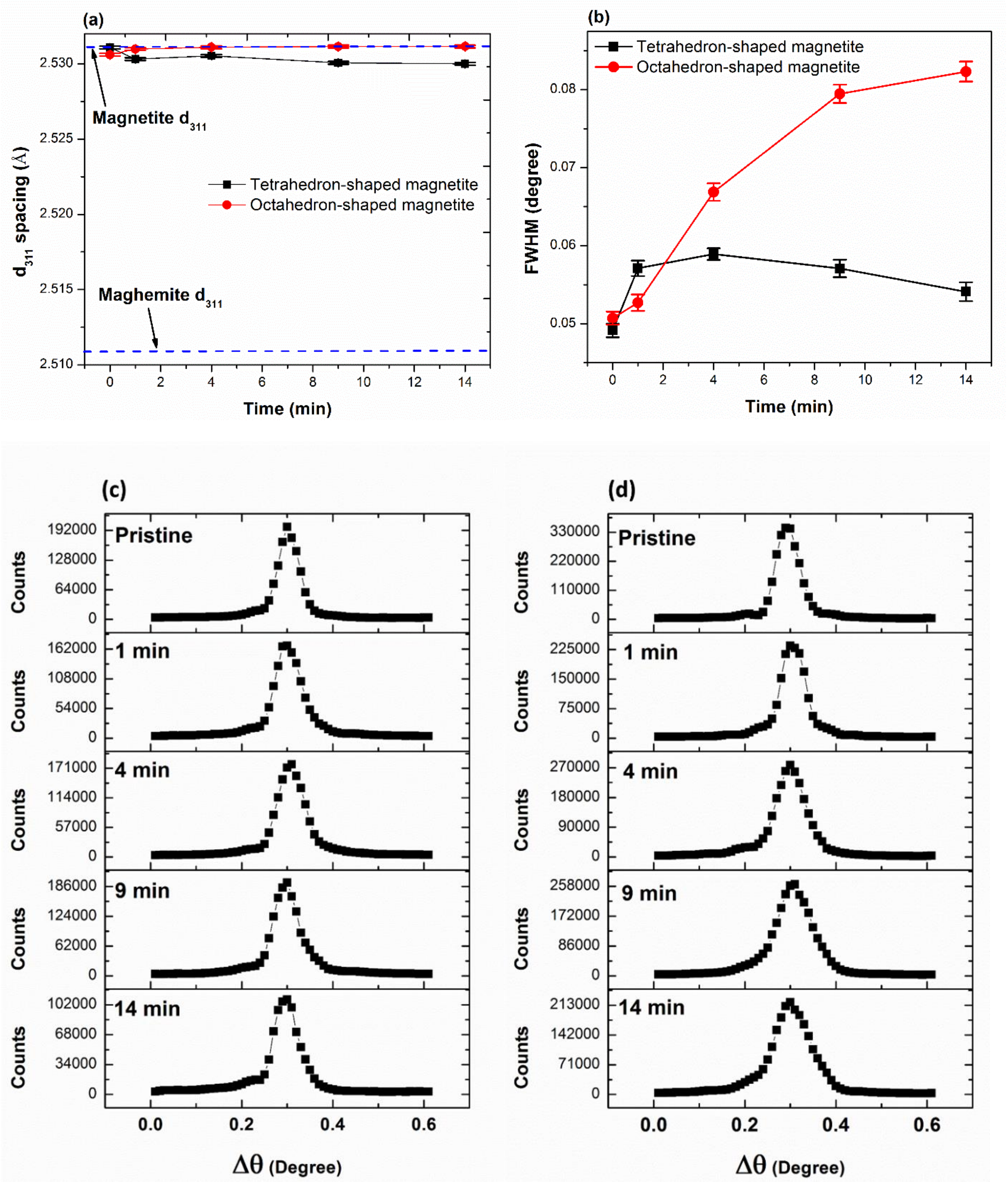

Supplementary Figure 2. Changes in magnetite d_{311} spacing and FWHM (°) of the magnetite (311) Bragg peak as a function of reaction time in a 0.1 M HCl solution. Dashed blue lines in (a) indicate the reported d_{311} values of magnetite and maghemite, respectively[1]. The error bars of the d_{311} values represent the standard deviations determined from multiple measurements. Error bars of the FWHM values are the standard deviations from peak fitting using a Gaussian function. Rocking scan curves of (c) the tetrahedron- and (d) octahedron-shaped magnetite.

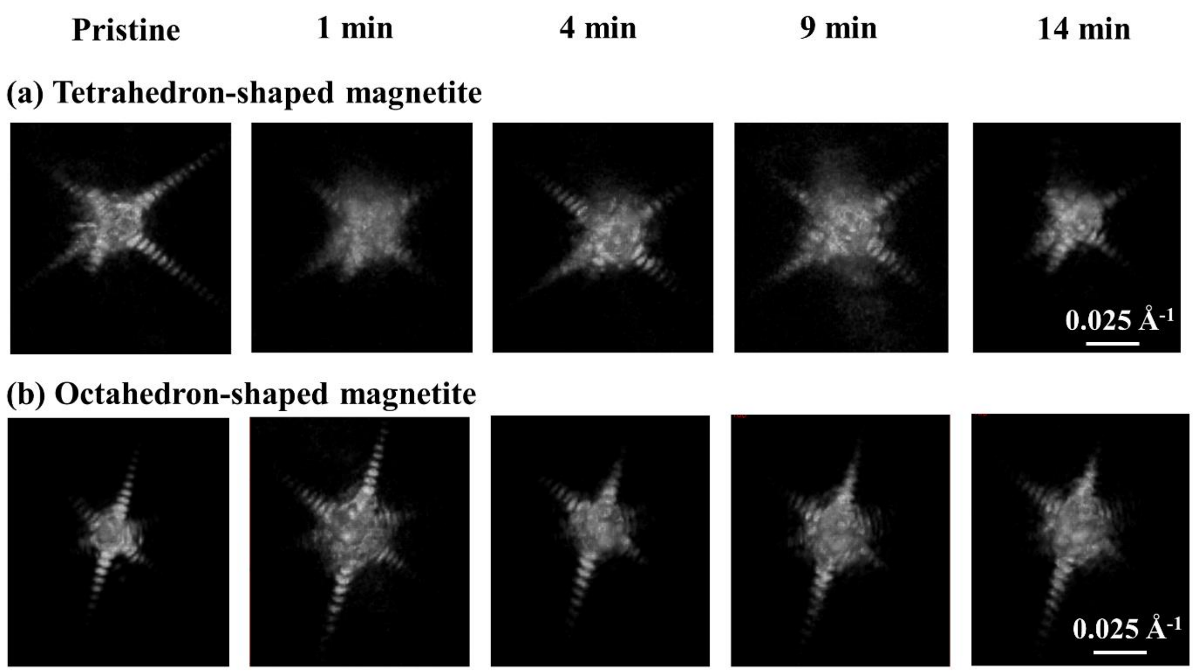

Supplementary Figure 3. **3D Bragg coherent diffraction patterns from the magnetite (311) plane**. (a) the tetrahedron- and (b) octahedron-shaped magnetite reacted in 0.1 M HCl for total of 14 min. Images were cropped to the same size to highlight the coherent Bragg pattern at the center of the detector, while the original detector area is larger.

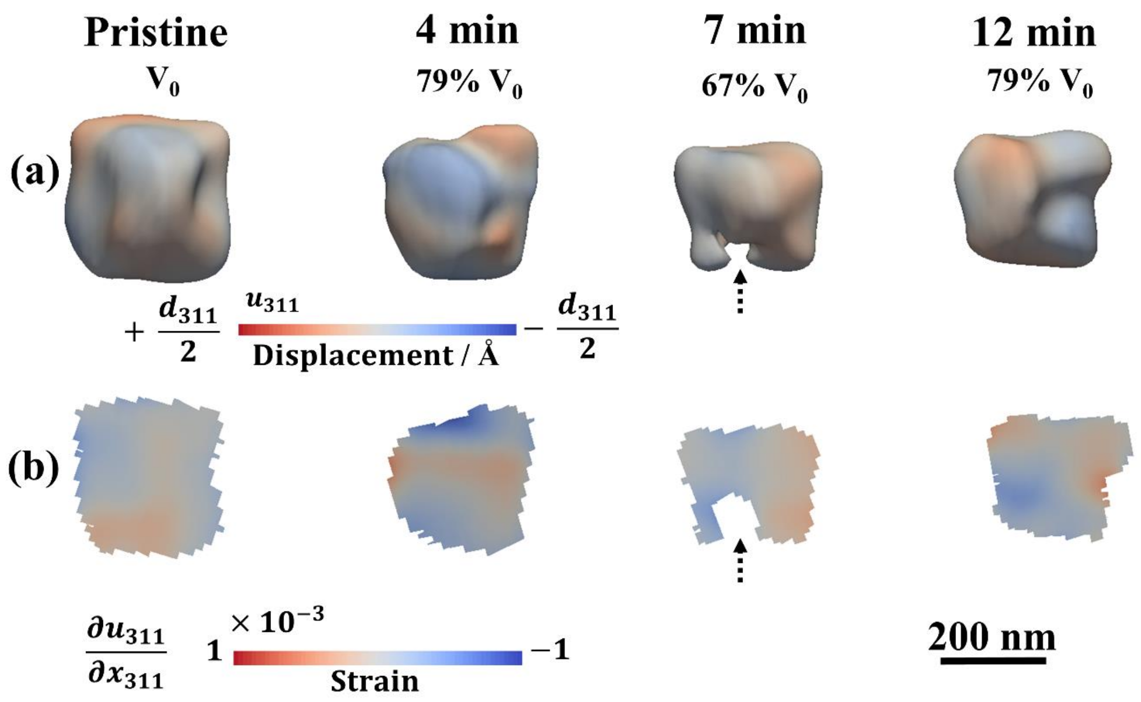

Supplementary Figure 4. **Dissolution of a magnetite crystal in a 0.1 M HCl solution for 12 min.** (a) 3D volume view at a 30% amplitude threshold. The displacement along the magnetite [311] direction was projected on the isosurfaces. (b) Cross-sectional views of the internal strain field.

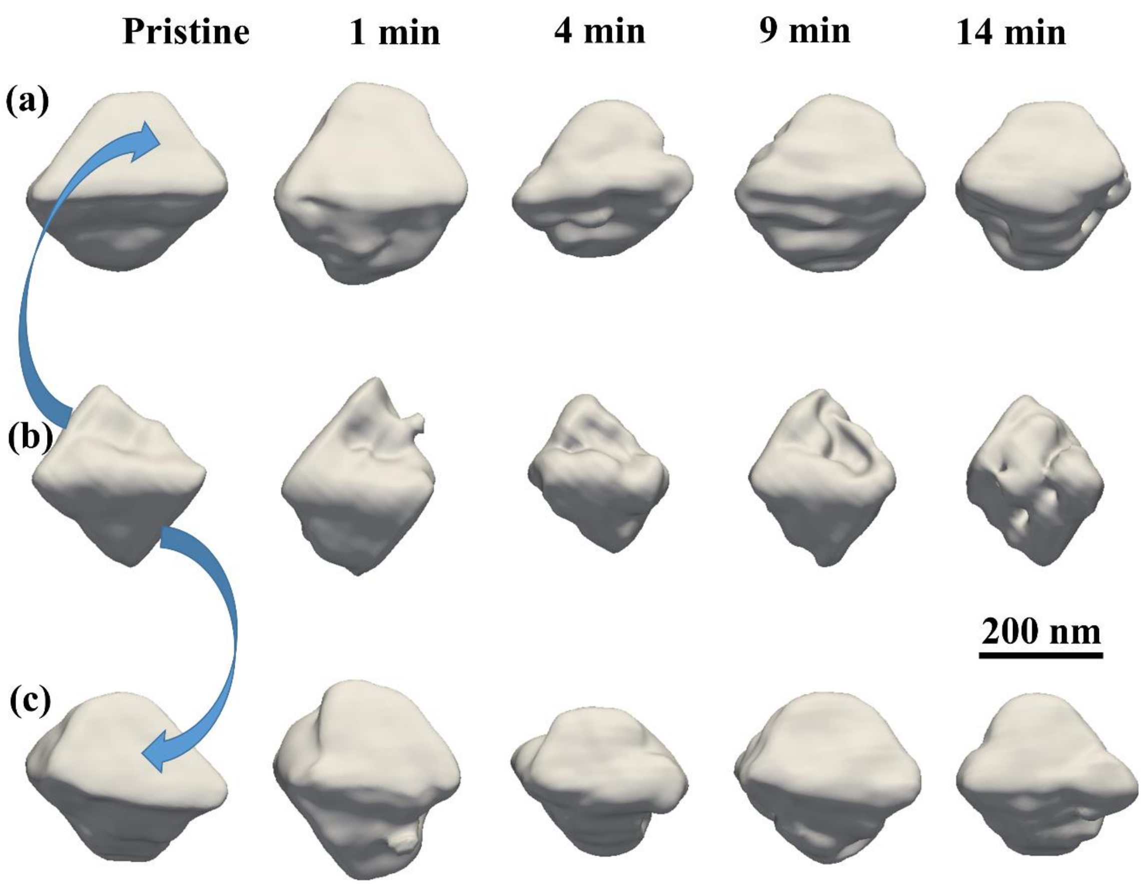

Supplementary Figure 5. **The octahedron-shaped magnetite shown in Fig. 2 viewed from three different perspectives.** (a) and (c) are created by rotating (b) in two opposite directions horizontally in order to highlight a pair of embedded smooth surfaces indicated by the blue arrows. The threshold level is at 30% of the amplitude.

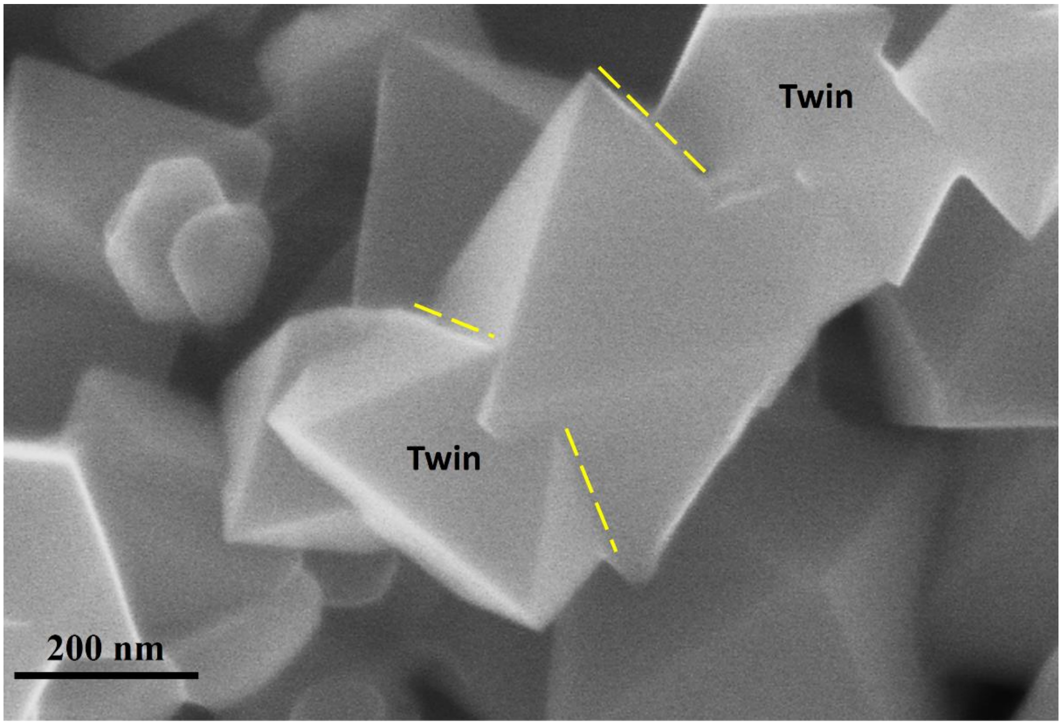

Supplementary Figure 6. **SEM images of twinned magnetite crystals found in the same set of sample used for BCDI measurements (not the crystal imaged by BCDI).** Three crystals were connected by sharing two (111) planes. The yellow dashed lines indicate the shared interfaces.

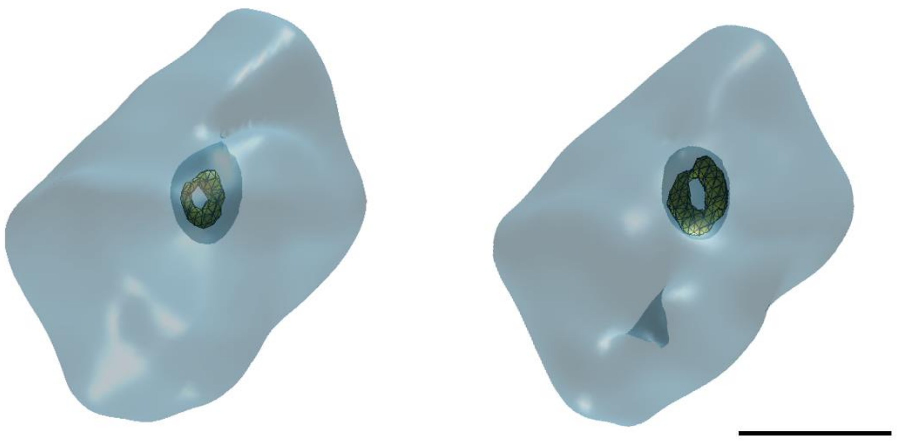

Supplementary Figure 7. Extracted dislocation loop in two additional BCDI measurements of the same magnetite crystal shown in Fig. 2 after 1 min of reaction in a 0.1 M HCl solution. Scale bar presents 100 nm.

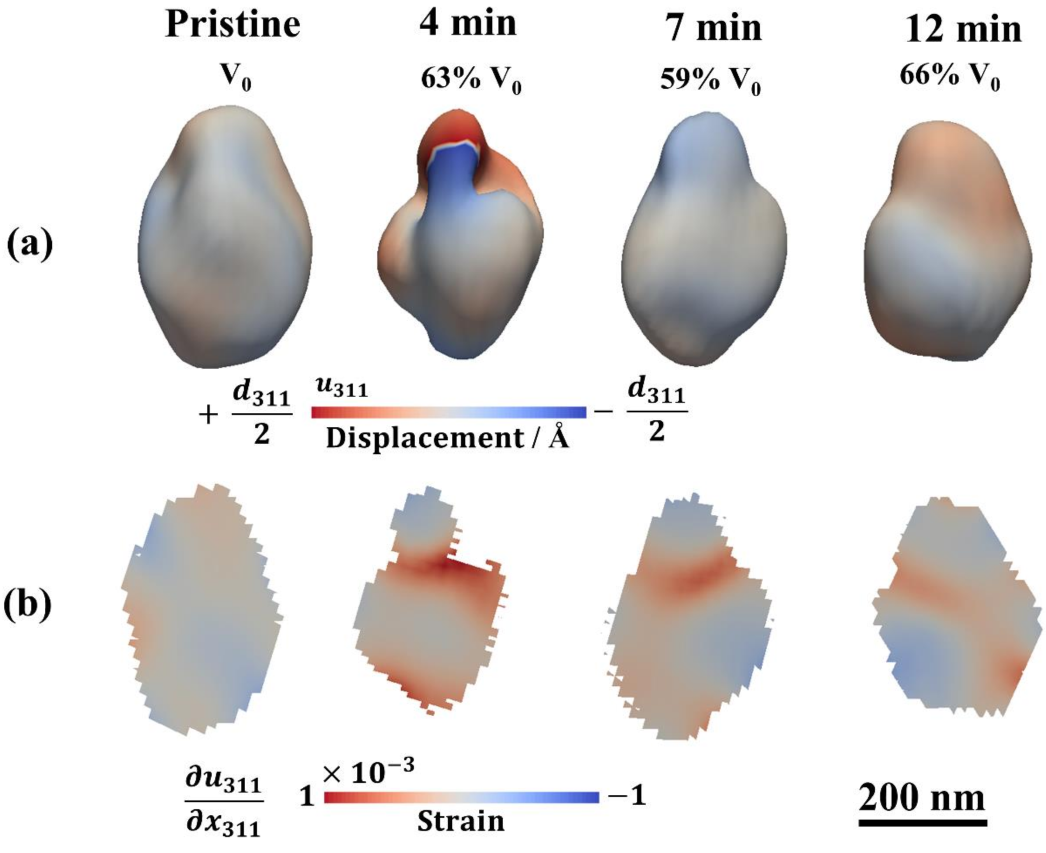

Supplementary Figure 8. Dissolution of the magnetite crystal showed in Fig. 4d. The crystal was reacted in a 0.1 M HCl solution for total of 12 min. Line dislocation was observed after 4 min of reaction and disappeared thereafter. (a) 3D morphology viewed at a 30% amplitude threshold. The displacement along the [311] direction were projected on the isosurfaces. (b) Cross-sectional views of the internal strain.

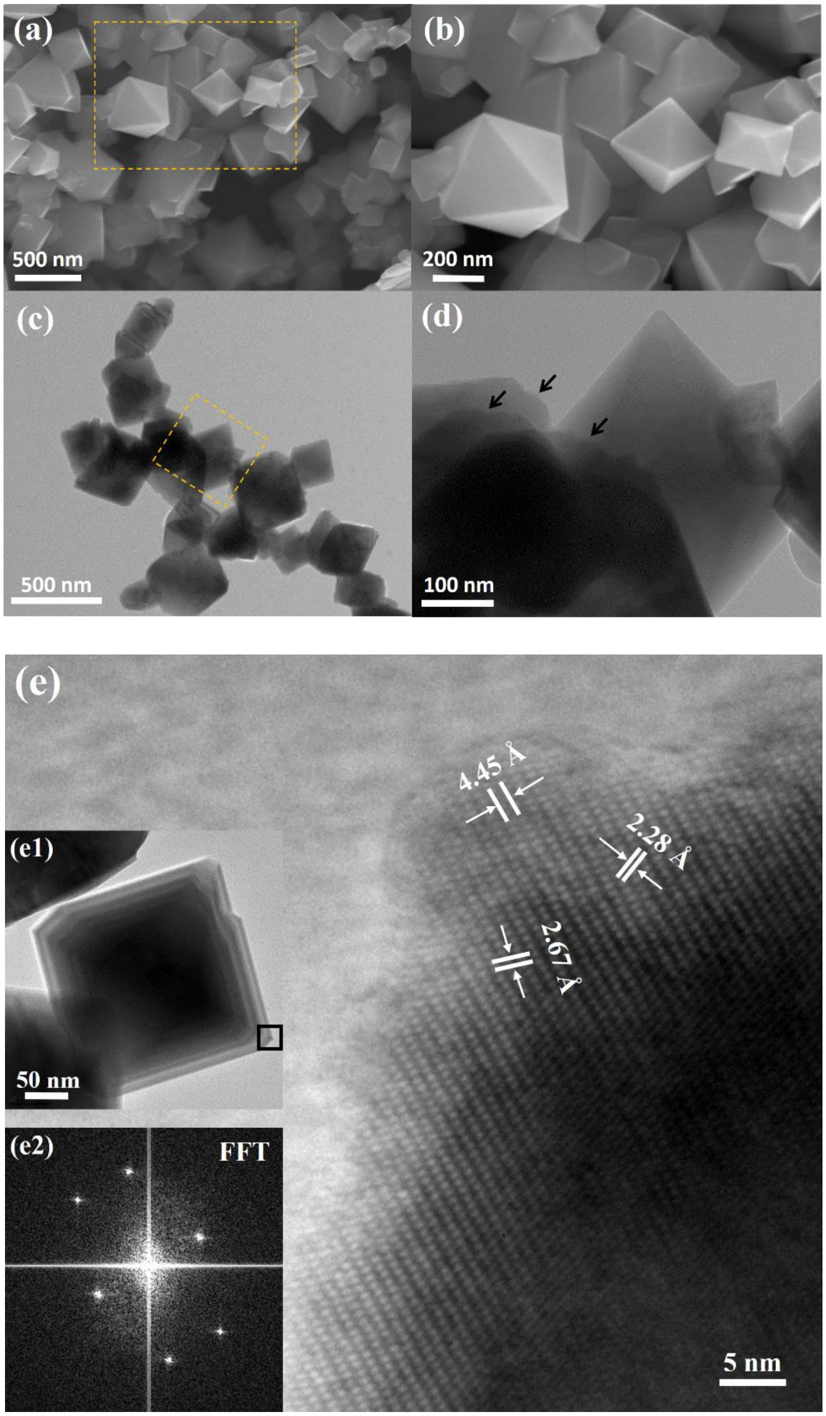

Supplementary Figure 9. **Electron microscopy images of magnetite crystals reacted in a 0.1 M HCl solution.** (a, b) SEM images of magnetite crystals obtained after the BCDI experiments, where the crystals have been reacted in acid for total of 14 min. (c, d, e) TEM images of separately prepared magnetite crystals reacted in 0.1 M HCl solution for 14 min. (e1) A TEM image of a magnetite crystal whose magnified view of one corner (box) is showed in (e).The Fourier transformed pattern of (e) is showed as (e2).

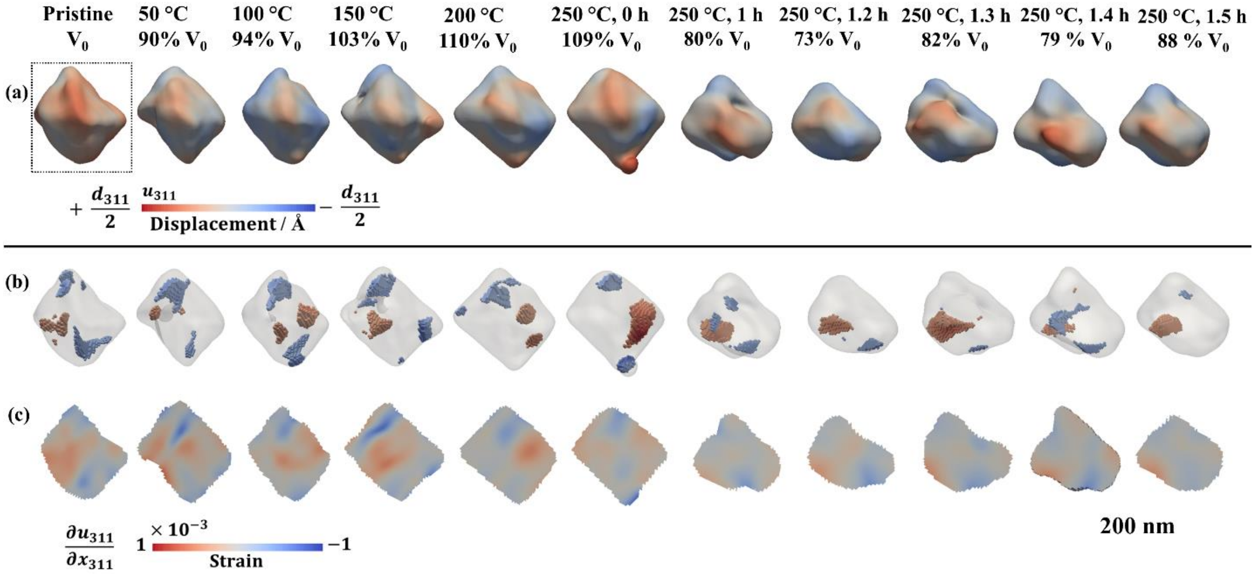

Supplementary Figure 10. **In situ heating experiment of an octahedral magnetite crystal up to 250 °C in air.** (a) 3D images at a 30% amplitude threshold with lattice displacements along the [311] direction projected on the isosurfaces. (b) 3D structure of compressive (blue, strain < -0.00035) and tensile (red, strain > 0.00035) strains. (c) Cross-sectional views of the internal strain field within the dashed line box in (a).

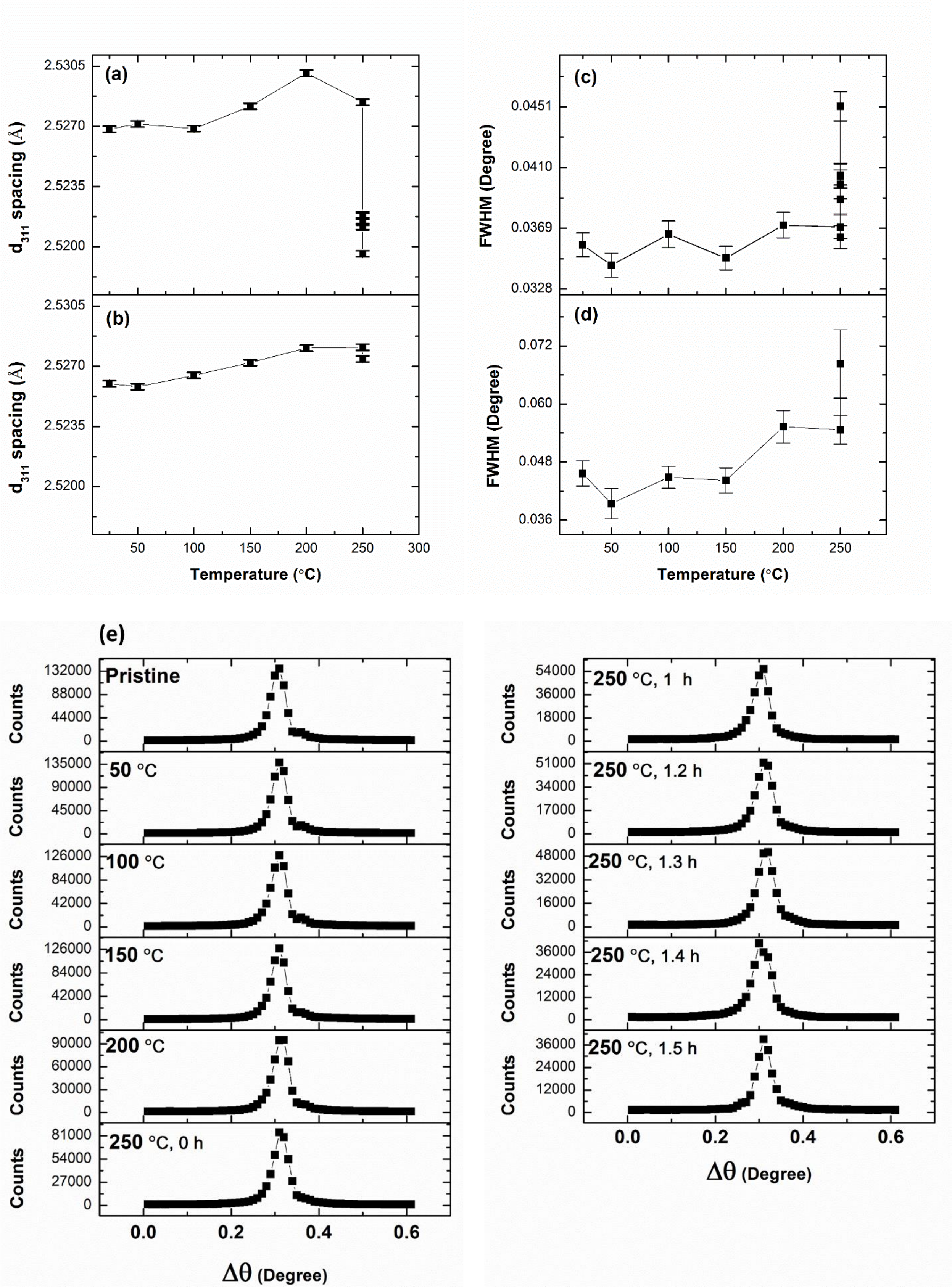

(a)
(b)
(c)
(d)
(e)
d_{311} spacing (Å)
2.5305
2.5270
2.5235
2.5200
FWHM (Degree)
0.0451
0.0410
0.0369
0.0328
0.072
0.060
0.048
0.036
50
100
150
200
250
300
Temperature (°C)
Counts
Pristine
50 °C
100 °C
150 °C
200 °C
250 °C, 0 h
250 °C, 1 h
250 °C, 1.2 h
250 °C, 1.3 h
250 °C, 1.4 h
250 °C, 1.5 h
132000
88000
44000
0
135000
90000
45000
126000
84000
42000
90000
60000
30000
81000
54000
27000
36000
18000
51000
34000
17000
48000
32000
16000
24000
12000
0.0
0.2
0.4
0.6
Δθ (Degree)

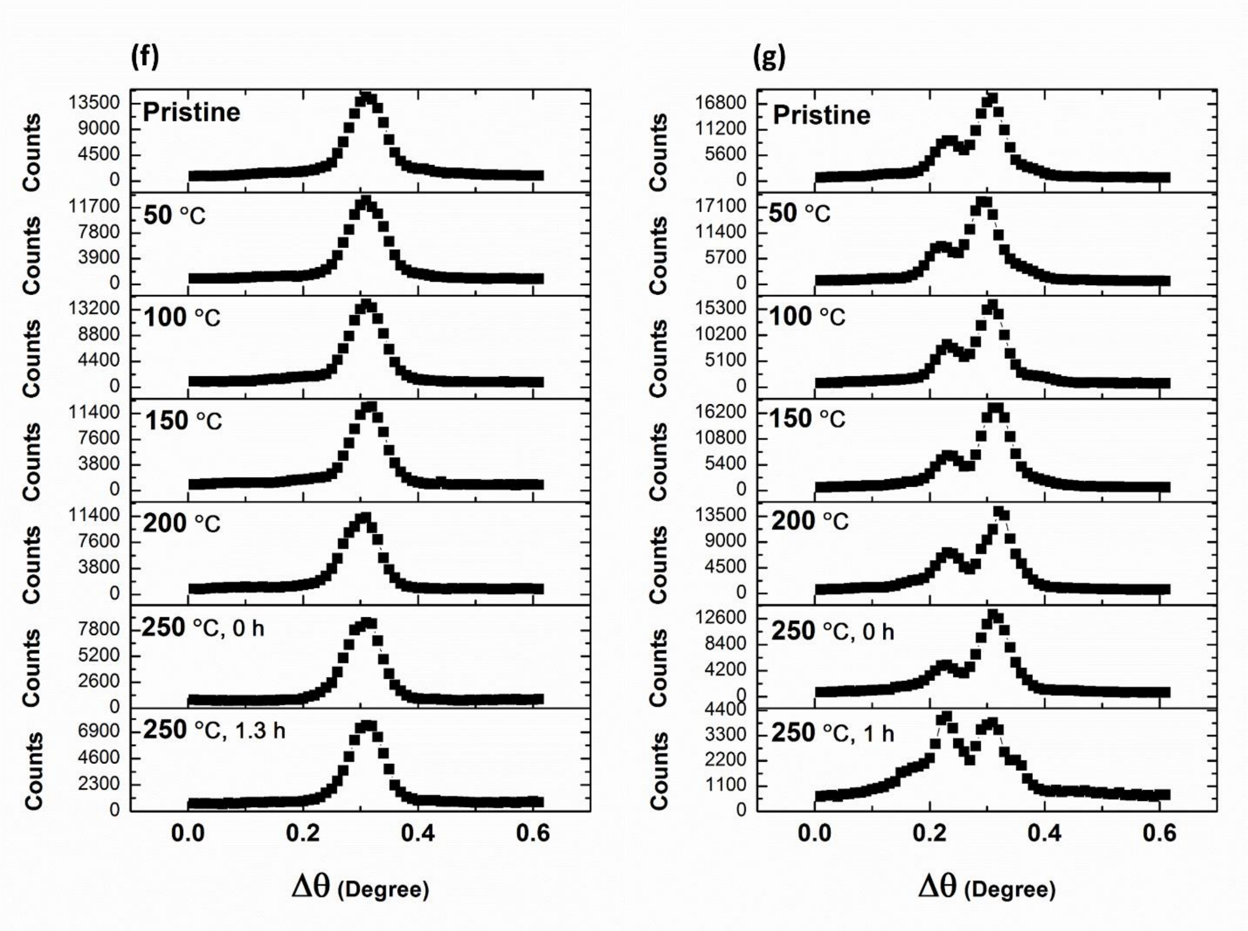

Supplementary Figure 11. **Magnetite d_{311} spacing (Å) and FWHM (°) of the (311) Bragg peak during thermal heating.** (a, c) and (b, d) were data of the two magnetite crystals shown in Fig. 5a and Fig. 5d, respectively. Error bars of the d_{311} spacing values represent the standard deviations determined from multiple measurements. Error bars of FWHM values are the standard deviations from peak fitting using a Gaussian function. (e), (f), and (g) are the rocking scan curves of the thermal heated magnetite shown in Fig. 5a, Fig. 5d, and Fig. 6, respectively.

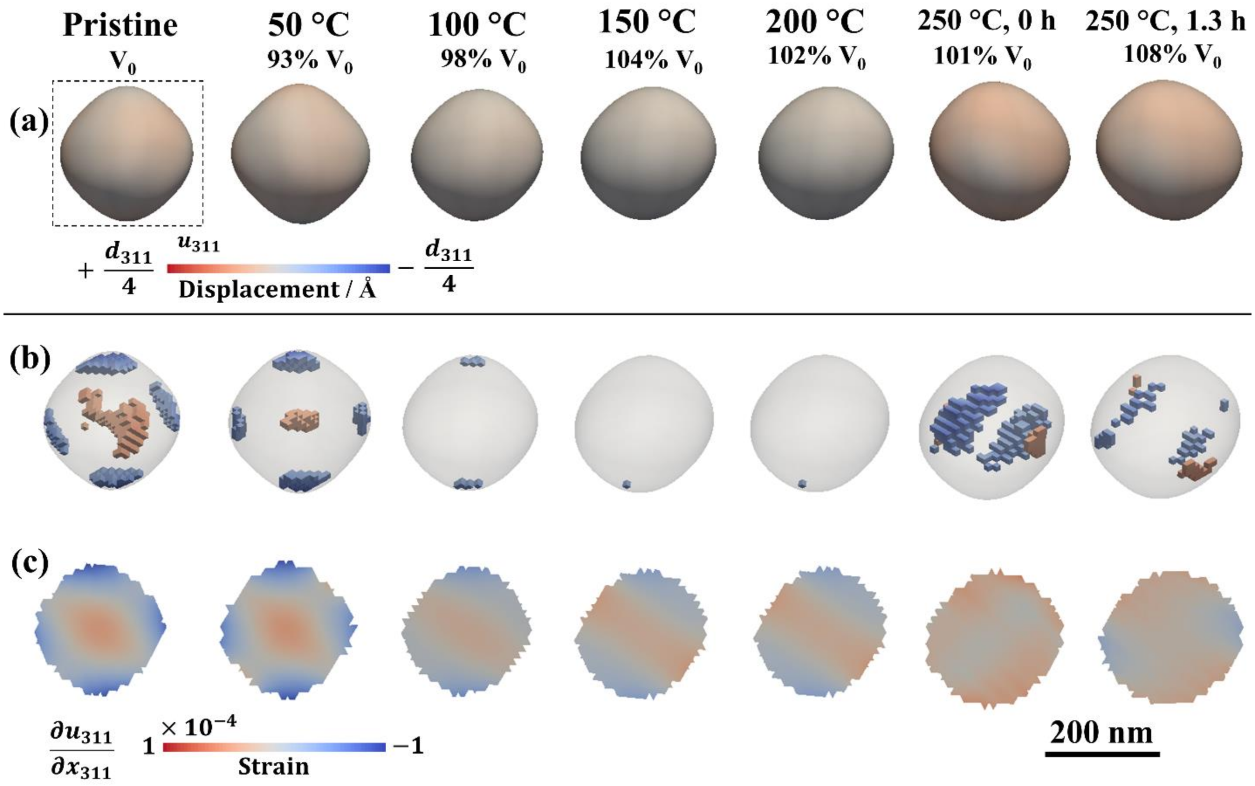

Supplementary Figure 12. **In situ heating of a magnetite crystal up to 250 °C in air.** (a) 3D volume views at a 30% amplitude threshold with lattice displacements along the [311] direction projected on the isosurfaces. (b) 3D strain structure of compressive (blue, strain < -3.5E-05) and tensile (red, strain > 3.5E+05) strains. (c) Cross-sectional views of the internal strain field at the dashed line box in (a).

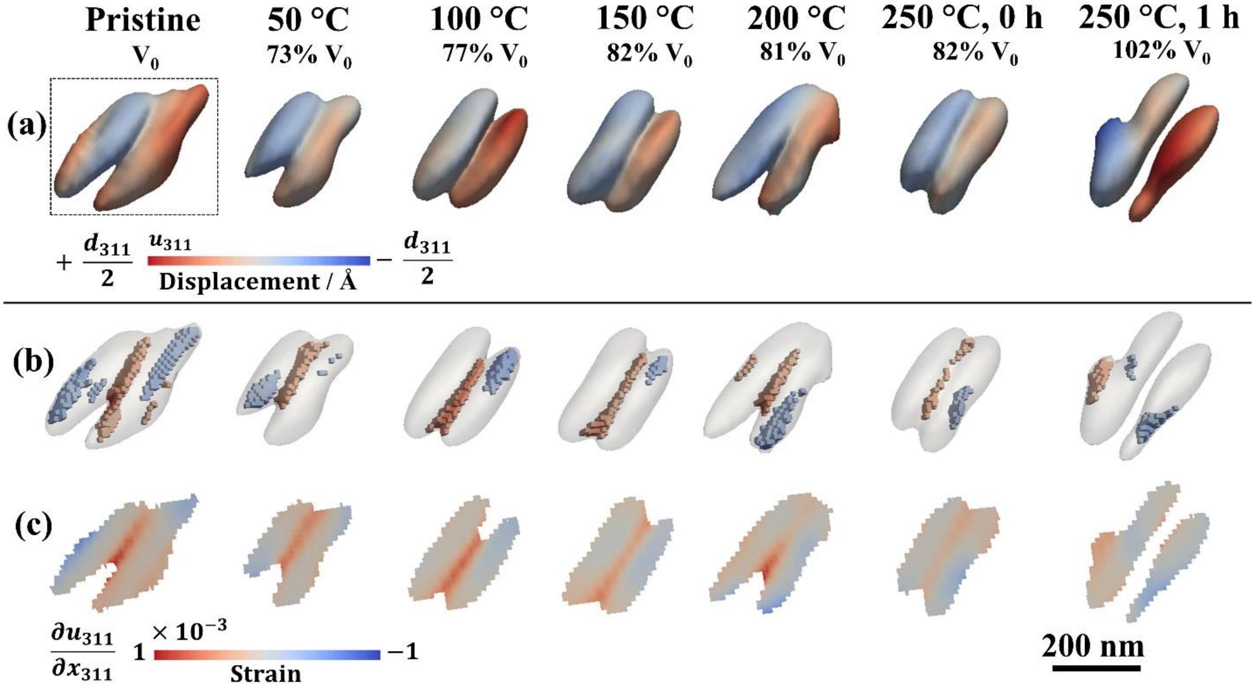

Supplementary Figure 13. **In situ heating of a twinned magnetite crystal up to 250 °C in air.** (a) 3D volume views at a 30% amplitude threshold with lattice displacements along the [311] direction projected on the isosurfaces. (b) 3D strain structure of compressive (blue, strain < -0.00035) and tensile (red, strain > 0.00035) strains. (c) Cross-sectional views of the internal strain field at the dashed line box in (a).

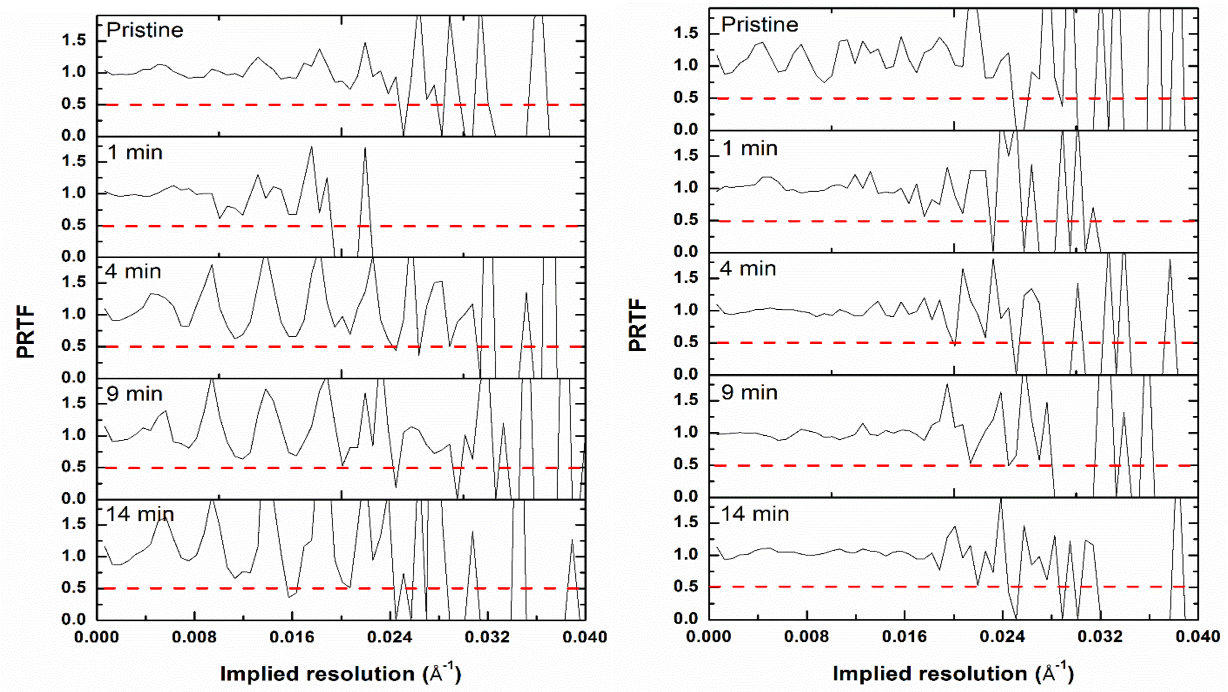

Supplementary Figure 14. **Implied resolutions determined from the phase retrieval transfer function (PRTF).** Data included the tetrahedron- and octahedron-shaped magnetite crystals during oxidative dissolution showed in Fig. 1 and Fig. 2, respectively.

Supplementary Table 1. **Determination of resolution from PRTF function**. The conservative estimation of the resolution is given by the frequency at which the PRTF reaches a value of 0.5.

Sample	PRTF implied resolution of tetrahedron-shaped magnetite ($Å^{-1}$)	PRTF implied resolution of octahedron-shaped magnetite ($Å^{-1}$)
Pristine	0.0247	0.0248
1 min	0.0191	0.0230
4 min	0.0311	0.0249
9 min	0.0242	0.0279
14 min	0.0242	0.0244
Average	0.0246 ± 0.0043	0.0250 ± 0.0018
Average in nm	26.1 ± 4.1	25.2 ± 1.6

Supplementary References

1. Gorski, C. A., Scherer, M. M. Determination of nanoparticulate magnetite stoichiometry by Mossbauer spectroscopy, acidic dissolution, and powder X-ray diffraction: A critical review. *Am. Mineral.* **95**, 1017-1026 (2010).
